# Recovery of woody plant species richness in secondary forests in China: a meta-analysis

**DOI:** 10.1038/s41598-017-10898-7

**Published:** 2017-09-06

**Authors:** Xiaofei Liu, Xuehua Liu, Andrew Skidmore, Claude Garcia

**Affiliations:** 1 State Key Joint Laboratory of Environmental Simulation and Pollution Control, and School of Environment, Tsinghua University, Beijing, 100084 China; 2 ITC, University of Twente, 7500 Enschede, AE The Netherlands; 3 Department of Environmental Science, Macquarie University, NSW 2109 Sydney, Australia; 4 ForDev Group, Department of Environmental Systems Science, Swiss Federal Institute of Technology (ETH), CH-8092 Zurich, Switzerland; 5 Research Unit Forests and Societies, Centre International de Recherche Agronomique pour le Développement (CIRAD), F-34392 Montpellier, France

## Abstract

There is considerable uncertainty concerning changes in plant diversity of Chinese secondary forests, particularly with respect to diversity recovery following anthropogenic disturbance. Here we present a meta-analysis of the recovery of woody plant species richness in secondary forests in China, with nearby primary forests as a reference. A total of 125 pairs of secondary-primary forest data reported in 55 publications were identified across China. We analyzed the data by region and logging history to examine their influences on secondary forest recovery. Our results indicated that the woody plant richness of secondary forests in China was close to fully recovered when compared to the primary forest, with the recovery ratio being 85–103%. Higher recovery ratios were observed in central, northeast and southwest China, with lower recovery ratios seen in east, south and northwest China, and the recovery in central China significantly reached the primary forests (reference) level. Concerning logging histories, the recovery ratios showed two peak values, with one at 21–40 years after clear cutting and the other at 61–80 years. We reveal the fundamental recovery patterns of woody plant species richness in secondary forests in China. These patterns provide information for the sustainable management of secondary forest resources.

## Introduction

The future of forests in the Anthropocene depends on the equilibrium between competing processes of forest recovery and human deforestation^1^. Recently, the focus of biological conservation has shifted to the scientific question of how the relationship between people and nature are viewed, and one of the key ideas is the ecosystem’s ability to recover from disturbance^2^. Ecosystem recovery following anthropogenic disturbances is increasingly occurring worldwide, either naturally or induced by human^3, 4^. In these ecosystems the estimation of biodiversity recovery is critical^3^ and the loss of biosphere integrity is one of the planetary boundaries that has been crossed^5^. The rate of global forest area net loss has slowed and the naturally regenerated forest has accounted for 69% of the forest cover^6^. However, considerable uncertainty remains about recovery in secondary forests^7^. Thus, a fundamental understanding of what has changed while forests and biodiversity recover following disturbance is of key importance for promoting conservation and further research on mechanism of recovery.

In highly diverse ecosystems, the function of any species lost to a disturbance can be replaced by other functionally similar species, hence biodiversity is thought to increase ecosystem resilience^8^. Resilience could be defined as the ability to recover after disturbance^7, 9^ and biodiversity has been used as an indicator of resilience^10–12^. Species richness, a measure of biodiversity, is widely used in meta-analyses for assessing recovery of ecosystems^10, 13, 14^. Recovery of species richness varies in different environment and it requires a considerable amount of time to recover to the reference level^4^ or may never become fully recovered^3^.

In China, secondary forests represent 57% of the forest resources^6^, covering roughly 121600 KHa^15^. In the 20^th^ century, forest cover in China fell to a historical low of less than 10 % by 1949, before it recovered to nearly 20 % by the end of the century^16^. The present forest ecosystems have been effectively protected since China has implemented forest-protection policies, and secondary forests play a pivotal role in conserving biodiversity in China^17^. Nevertheless, we lack a clear picture of biodiversity recovery patterns in secondary forests on a national scale.

The methods of meta-analysis were introduced in ecology in the early 1990s and provide a powerful and informative set of tools for summarizing the results of studies on the same topic^18^. Remote sensing and trait databases such as TRY are powerful tools for biodiversity monitoring and conservation planning at large scales^4, 19^. However, for identifying individual species, the meta-analysis can be rather effective^3, 10, 13^. No meta-analysis on the plant richness recovery in secondary forests in China has been undertaken previously.

In this study, we aim to address two fundamental questions by conducting a meta-analysis of published information. First, in China, to what extent has the woody plant richness of secondary forests recovered from anthropogenic disturbance? Second, what are the spatial and temporal patterns of woody plant richness recovery in secondary forests in China? We hope this study could provide information for the sustainable management of secondary forest resources.

## Results

### Characteristics of data sets

**Figure 1:**
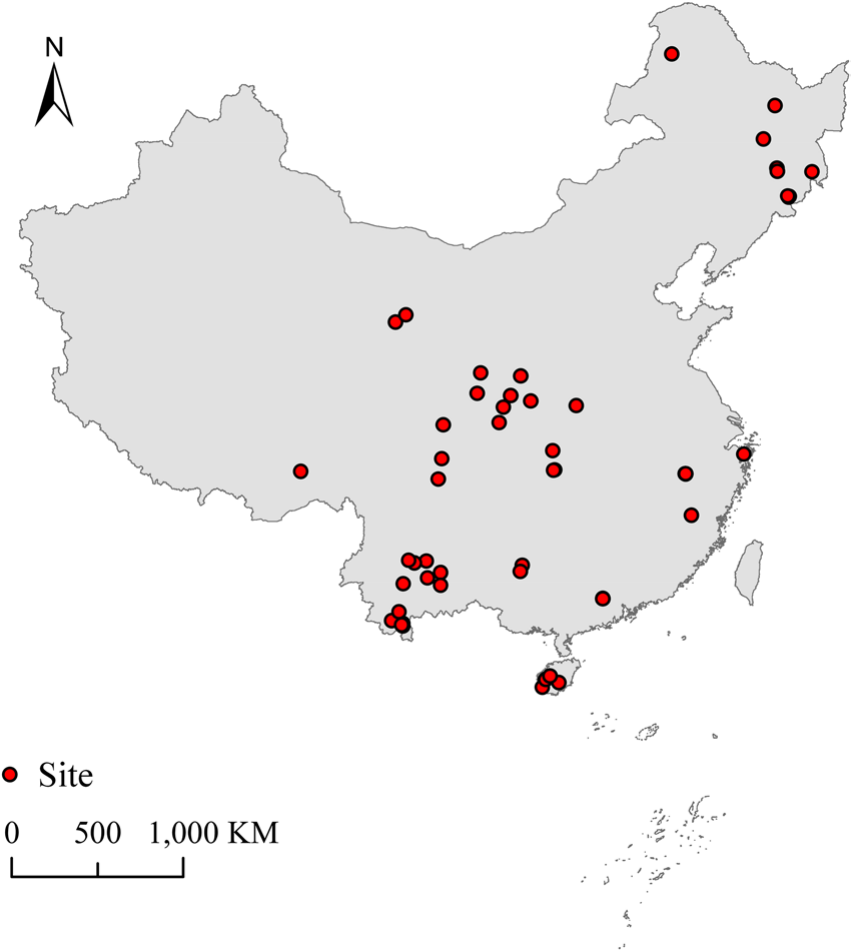
Locations of all studies included in this meta-analysis. The majority of locations were in the Changbai Mountains in Jilin Province, Qinling Mountains in Shaanxi Province, Ailao Mountains in Yunnan Province and Bawangling in Hainan Province. This figure was generated by ArcGIS 10.3.

**Figure 2:**
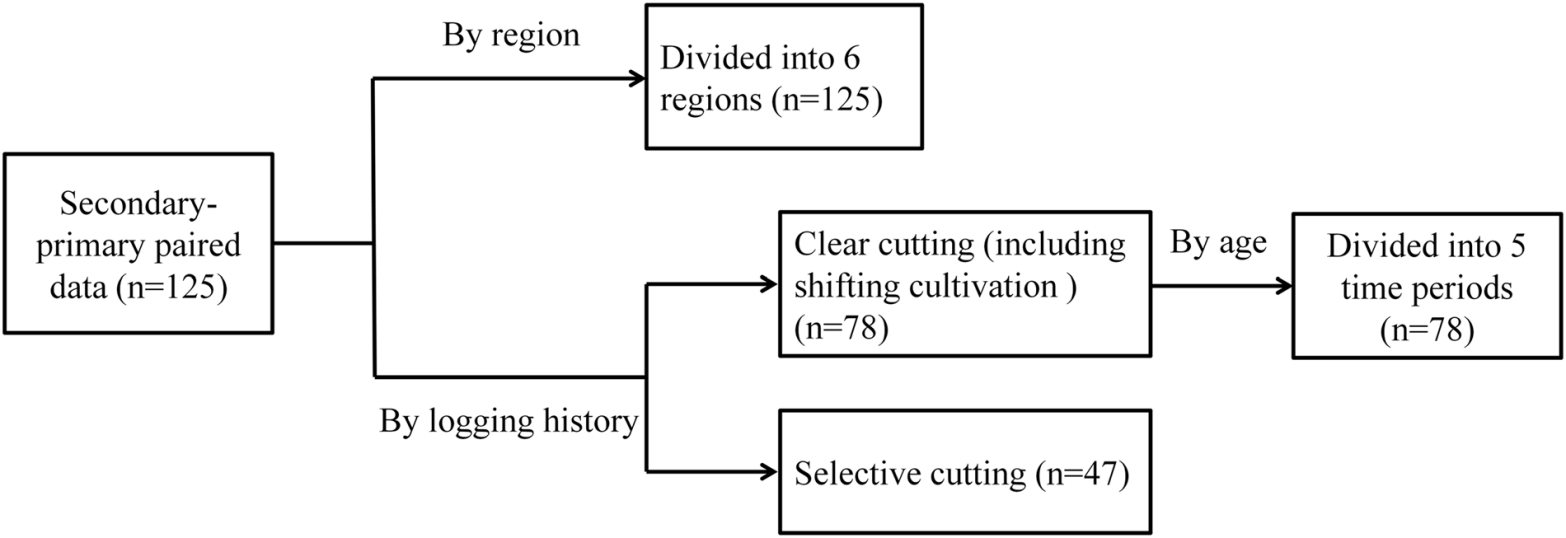
The workflow of preparing data sets. The data were sorted by region and logging history. **n** is the number of data pairs.

Our data set covered 55 publications, which provided 125 second-primary paired data across China (Fig. 1). We sorted the data by region and logging history (Fig. 2). The data sorted by region contained 6 regions, according to their geographic locations and administrative divisions in China: northeast, northwest, central, east, southwest and south China. Logging histories contained two types of logging: clear cutting (including shifting cultivation) and selective cutting. Furthermore, the clear cutting data were divided into 5 time periods reflecting the forests’ age: <=20 years, 21–40 years, 41–60 years, 61–80 years and >80 years.

**Figure 3:**
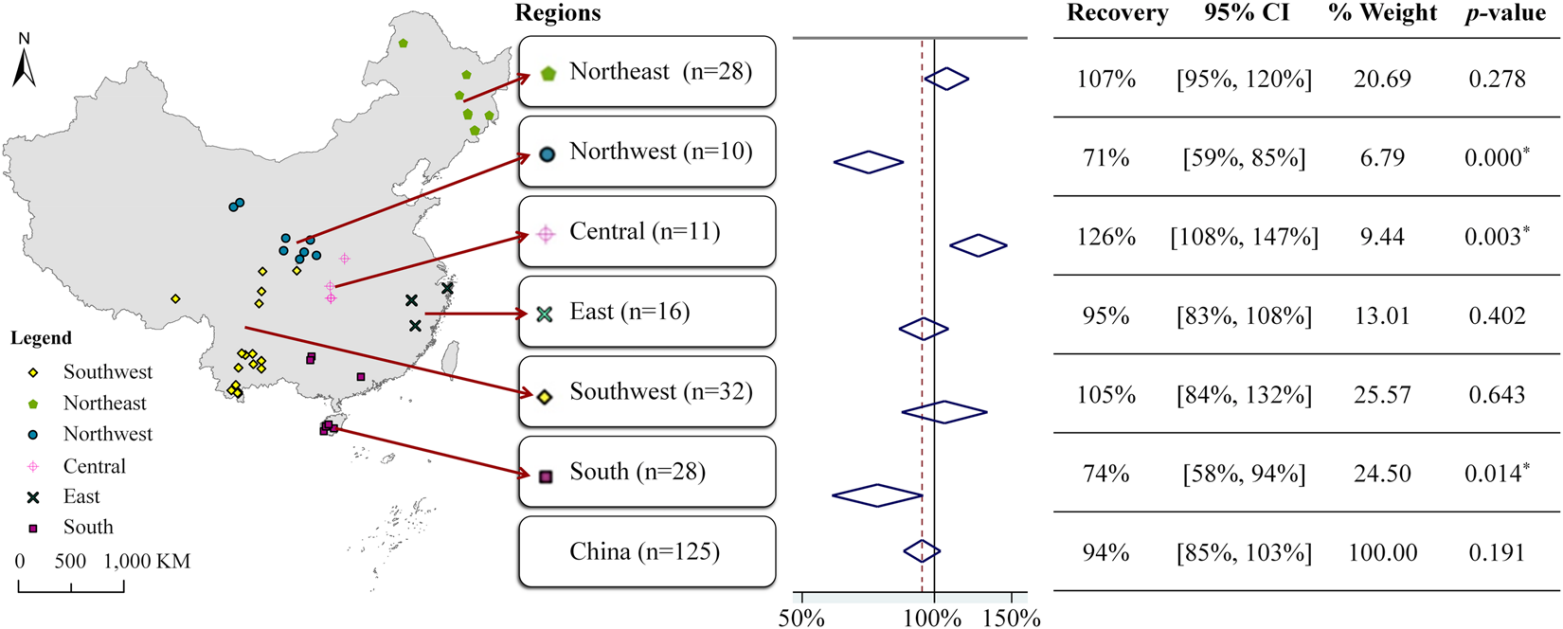
Woody plant richness recovery of secondary forests in different regions. The area was divided into 6 broad geographic regions: northeast, northwest, central, east, southwest and south China. **n** is the number of data pairs in each region. The forest plot uses diamonds to summarize effect sizes (recovery) and 95% CIs of random-effect models for each of the 6 regions. The vertical dashed red line represents the estimated mean value and the vertical black line indicates 100% recovery. The *p*-value is for the test of recovery *R* = 100%. The map was generated by ArcGIS 10.3.

**Figure 4:**
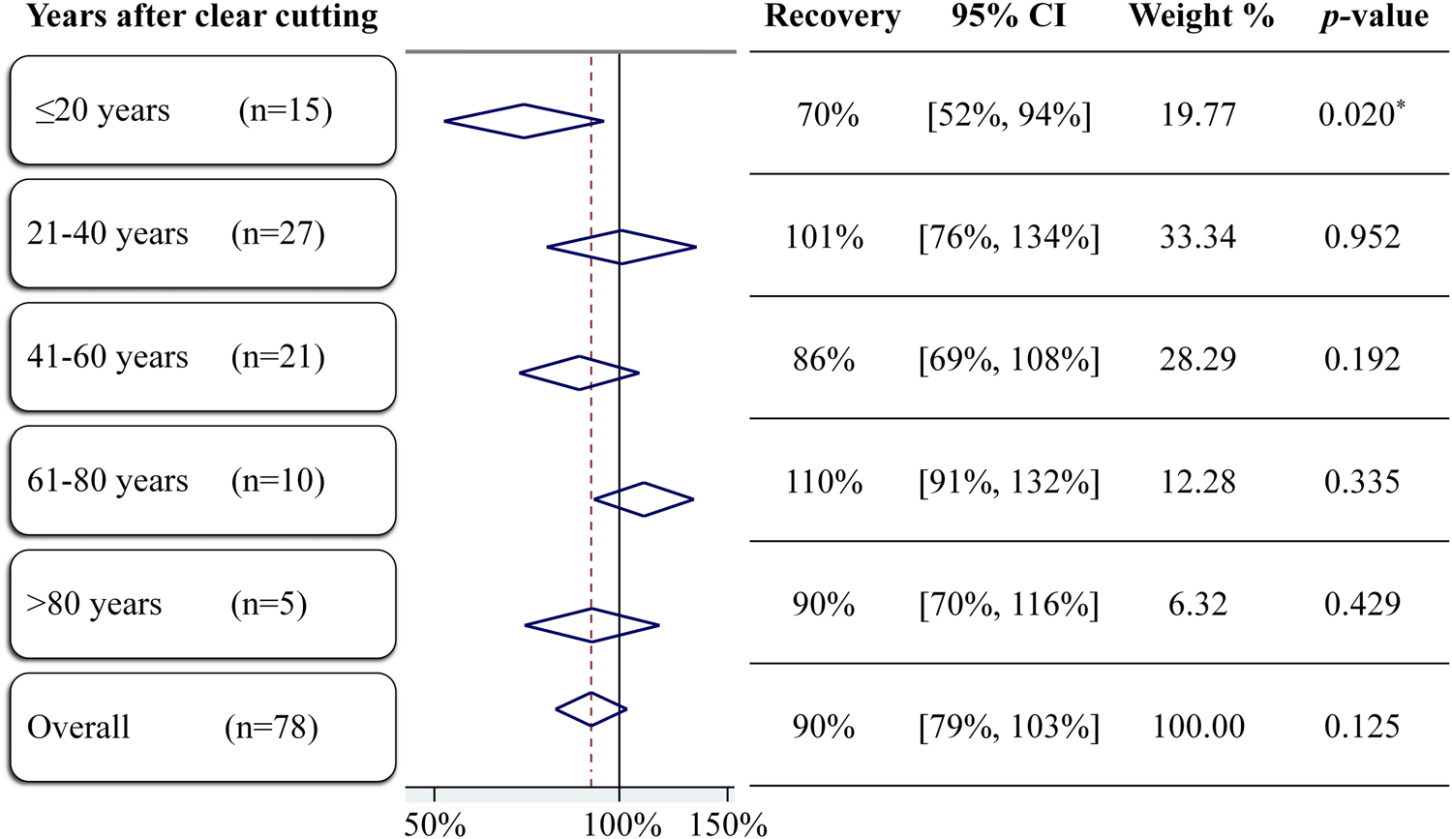
Woody plant richness recovery of secondary forests after clear cutting. **n** is the number of pairs of data in this time period. The forest plot uses diamonds to summarize effect size and 95% CIs of random-effect models for 5 time periods. The vertical dashed red line represents the estimated mean value and the vertical black line shows 100% recovery. The *p*-value is for the test of 100% recovery.

Among 125 paired data, 28 pairs were in northeast China, 10 pairs in northwest, 11 pairs in central, 16 pairs in east, 32 pairs in southwest and 28 pairs in south China (Fig. 3). In terms of logging history, 78 pairs described the secondary forests after clear cutting and 47 pairs were after selective cutting. With respect to the time after clear cutting, 15 pairs were within 20 years after clear cutting, 27 pairs after 21–40 years, 21 pairs after 41–60 years, 10 pairs after 61–80 years and 5 pairs were of forests cut more than 80 years prior (Fig. 4). The overall woody plant richness recovery of the secondary forests in China was 94 % (mean effect size), with a 95% CI of [85%, 103%] from the random-effects model, compared with nearby primary forests.

There was significant heterogeneity in the studies for overall secondary forests, for selective cutting and for clear cutting (*p(Q-df)* < 0.05, Table 1). Their *I* ^2^ were 78.6%, 64.2% and 82.8% respectively, indicating that a substantial amount of between-study variance could be attributed to real differences in true effect size of studies. The southwest and south geographic regions showed significant heterogeneity (*p(Q-df)* < 0.05), with the south region being the largest according to the *Tau* ^2^ value. There was also significant heterogeneity in the three time periods <=20 years, 21–40 years and 41–60 years after clear cutting (*p(Q-df)* < 0.05).

**Table 1:** Heterogeneity of the data sets.

	*n*	*p*(Q-*df*)	*I* ^2^	*Tau* ^2^
**Regions**				
Overall	125	0.000	78.6%	0.2195
Northeast	28	0.656	0.0%	0.0000
Northwest	10	0.604	0.0%	0.0000
Central	11	0.383	6.4%	0.0042
East	16	0.244	18.3%	0.0123
Southwest	32	0.000	84.6%	0.3291
South	28	0.000	89.4%	0.3536
**Logging histories**				
Selective cutting	47	0.000	64.2%	0.1121
Clear cutting				
Overall	78	0.000	82.8%	0.2824
<=20 years	15	0.000	83.5%	0.2785
21–40 years	27	0.000	88.2%	0.4487
41–60 years	21	0.000	81.0%	0.2133
61–80 years	10	0.383	6.4%	0.0059
>80 years	5	0.942	0.0%	0.0000

The data were sorted by region and logging history. *n* is the number of data pairs. **Q-** ***df*** is the excess variation, which means that the part that will be attributed to differences in the true effects from study to study. A significant ***p(Q-df)*** value (*p* < 0.05) provides evidence that the true effects vary. ***I*** ^2^ is the ratio of true heterogeneity to total variance across the observed effect estimates. *Tau* ^**2**^ is the estimation for the variance of true effects.

We performed a publication bias identification and sensitivity analysis (Supplementary Information 1), and found insignificant publication bias in our dataset (n = 125, *p*  = 0.796), showing the robustness of meta-analysis results.

### Species richness recovery of secondary forests in different regions

In different regions, recovery of woody plant richness within secondary forests was different (Fig. 3). The recovery rate of secondary forests in central China (n = 11) was 126% with a 95% CI of [108%, 147%], significantly higher than those in other regions (*p* = 0.003), as 107% in northeast China (n = 28), 105% in southwest China (n = 32) and 95% in east China (n = 16). The recovery in northwest China (n = 10) was 71% with a 95% CI of [59%, 85%], significantly less than 100% (*p* = 0.000). The recovery in south China (n = 28) was 74% with a 95% CI of [58%, 94%], also significantly less than 100% (*p* = 0.014).

### Species richness recovery of secondary forests with different logging histories

Our analysis results evidenced that the recovery of woody plant richness varied with logging histories. The recovery of secondary forests after clear cutting (n = 78) was 90%, with a 95% CI of [79%, 103%], while the recovery of secondary forests after selective cutting (n = 47) was 100%, with a 95% CI of [88%, 113%].

The recovery of secondary forests after clear cutting showed two peak values (Fig. 4). The first one was after 21–40 years and the other was after 61–80 years. The recovery ratio of forests within 20 years was minimal and significantly less than 100% (*p* = 0.020), whereas all the estimated 95% CIs of other time periods covered 100% recovery.

## Discussion

### In China, to what extent has the woody plant species richness of secondary forests recovered from anthropogenic disturbance?

Our meta-analysis showed that, using primary forests as a reference, the woody plant richness of secondary forests in China was close to fully recovered, with the recovery ratio being 85–103% (Fig. 3). A possible reason is that China started implementing the Natural Forest Protection Program and Nature Reserves Construction Program, and both the area and quality of secondary forests have been largely improved^17, 20^. Zhang and Liang^21^ also explored recovery of forests in northeast China but focused on the forest biomass, and found it increased significantly between 2001 and 2010. By analyzing 55 independent publications across China, we show for the first time, on a national scale, that woody plant richness of secondary forests has recovered to similar values associated with primary forests in China.

The secondary forests in China are mainly caused by anthropogenic disturbance (e.g. logging and cultivation)^22^. In our meta-analysis, we only analyzed secondary forests that recover from anthropogenic disturbance. When filtering publications, we found that secondary forests caused by natural factors were in the minority. We excluded 3 publications where forests had regenerated following natural factors (two forest fires and one mudslide). In human modifed forest, besides species diversity, the forest biomass^7^, productivity as well as carbon pools^23^, nitrogen cycling^3^ are all altered. In the Anthropocene, shaping the transition of forests and promoting recovery and resilience of forests will become increasingly necessary in the coming decades^1, 2^.

### The spatial and temporal patterns of woody plant species richness recovery in secondary forests in China

The broad geographical regions obviously affected recovery. Higher species richness recovery ratios were found in central, northeast and southwest China compared with east, south and northwest China. Recovery in central China reached the significant reference level (*p* = 0.003), but northwest and south China significantly did not (*p* = 0.000 and *p* = 0.014), see Fig. 3. In the heterogeneity analysis, significant heterogeneity suggested that the difference in recovery was considerable. Even within one region, for example southwest and south regions, the richness recovery varied greatly (Table 1). We found that the forest type could lead to a difference in recovery. Of the 55 publications, 2 studies were conducted in pure coniferous forests. One was from spruce forests in the Qilian Mountains where *Picea crassifolia* Kom. was the dominant species. The other was from fir forests in Tibet and *Abies georgei* var. *smithii* was the dominant species. Since there was only 1 dominant tree species in these forests, the recovery ratios reached 100% more easily than those of broad-leaved forests or mixed forests. The heterogeneity may be also attributable to abiotic drivers such as soil fertility^24^, and biotic influences such as the distance from nearby primary forests^25, 26^, as well as the experienced disturbance and management^27^. Due to the limited sample size and lack of detail in the publications, our analysis could not distinguish the main causes among these abiotic and biotic factors. However, our goal here is to reveal the recovery patterns of plant species richness in secondary forests in China. Nevertheless, our research details the spatial recovery pattern on a national scale and provides useful information for forest restoration and management.

Our results indicated that within 20 years after clear cutting, the woody plant richness could not recover to the reference level (*p* = 0.020). In other time periods, statistics showed that they probably could reach the full recovery, as the 95% CIs contained 100% recovery. Martin *et al*.^13^ also suggested that in secondary tropical forest, the tree species richness could be recovered after about 50 years. However the plant richness seems not to continuously increase in secondary succession^13^. Our data were collected from 55 publications, and the secondary forests of these publications were across China with different logging history. On such time and space scales, the recovery showed two peak values, with one after 21–40 years and the other after 61–80 years, see Fig. 4. This is probably because species richness accumulates with pioneer species^10^, followed by a decrease caused by competition, and then the growth of shade-tolerant species resulting in the second explosion of species before stabilizing^28^. We provide supporting evidence that the restoration time strongly drives recovery in secondary forests^29^. The significant heterogeneity in those three time periods that less then 60 years may come from the different recovery rates^30^ or the subgroup setting^31^, which is the division of ages in this analysis. Despite the heterogeneity analysis reported here, our results concerning the time after clear cutting contributes insights into trends of secondary forest recovery.

### For future research

We used species richness as a measurement of biodiversity and found that species richness recovered well. However, species richness is not the only aspect to explore when addressing forest recovery. Phylogenetic diversity, species similarity, stand structure, functional traits composition, all these play a role in the resilience of the forest ecosystem. Comparisons between secondary forests and primary forests always show marked differences in community composition^13, 32, 33^, whereas the species composition takes far longer to recover than species richness^29^. Future work, incorporating a meta-analysis concerning species similarity between secondary forests and primary forests, could lead to deeper understanding of biodiversity alterations and further improve the assessment of forests recovery in China. Only if we can understand compositional shifts and the changes in functional groups during recovery process will we be able to reveal potential changes of the resilience of secondary forests in China.

## Conclusion

Using a meta-analysis approach, we have shown that the woody plant species richness of secondary forests in China was close to fully recovered when compared with primary forests, with recovery ratio being 85–103%. Higher recovery ratios were observed in central, northeast and southwest China, with lower recovery ratios seen in east, south and northwest China. Recovery in central China significantly reached reference level (*p* = 0.003), but secondary forests in the northwest and south significantly did not recover (*p* = 0.000 and *p* = 0.014). Additionally, within 20 years after clear cutting, the woody plant richness could not be fully recovered (*p* = 0.020), and the recovery ratios showed two peak values thereafter, with one after 21–40 years and the other after 61–80 years. These patterns provide information for the sustainable management of secondary forest resources. We suggest future research to incorporate a meta-analysis on species composition similarity between secondary forests and primary forests, which will improve the assessment of forest recovery in China and provide a deeper insight into resilience of secondary forests.

## Methods

### Study selection

We performed a search in Web of Science™ Core Collection for the timespan 2000 – 10^th^ January 2017 using the following search term: TOPIC = (second* forest*) AND TOPIC = (China OR Chinese) AND TOPIC = (diversity OR richness). This yielded 274 publications. For Chinese-language publications, we used the China Knowledge Resource Integrated Database (http://www.cnki.net/) for the same timespan and matching these term (in Chinese): TOPIC = (secondary forest) AND/OR TOPIC = (primary forest OR natural forest OR old forest) AND TOPIC=(diversity OR richness), TOPIC = (secondary forest) AND (Beijing OR Tianjing OR Shanghai OR Chongqing OR Hebei OR Shanxi OR Liaoning OR Jilin OR Heilongjiang OR Zhejiang OR Jiangsu OR Anhui OR Fujian OR Jiangxi OR Shandong OR Henan OR Hubei OR Hunan OR Guangdong OR Hainan OR Sichuan OR Guizhou OR Shaanxi OR Yunnan OR Qinghai OR Gansu OR Guangxi OR Xinjiang OR Tibet OR Inner Mongolia OR Ningxia OR Hong Kong OR Macao OR Taiwan), TOPIC = (secondary forest) AND (Qinling Mountains OR Taihang Mountains OR Hinggan Mountains OR Qilian Mountains OR Wuyi Mountains). This search resulted in approximately 800 publications including papers and theses. We also searched relevant Chinese books.

We then applied the following criteria to filter publications, and any publication not meeting all the criteria below were excluded:

1. The studies were carried out in China and must have been published after 2000. We excluded earlier studies because we only wanted to focus on recent recovery patterns of Chinese secondary forests.
2. For each study, both secondary forests and primary forests must have been surveyed, and the primary forests should be adjacent to the secondary forests.
3. The plant richness must have been reported as the original number of species observed instead of a calculated index.
4. The history of secondary forests must have been illustrated clearly. The secondary forests must be caused by human disturbance not natural factors. Clear cutting (including shifting cultivation) or selective cutting should have been identified and the time of logging should have been recorded.
5. The single standard sample area must have been larger than 20 m × 20 m.

These criteria reduced the initial number of publications to 55. Among 55 publications, 23 publications were in English and 32 were in Chinese, including 17 theses and 5 books (Supplementary Information 2).

### Data extraction

From the selected publications, we adopted the following criteria to extract pairs of secondary-primary data for calculating recovery ratios. (1) For calculating plant recovery, we chose only woody species and excluded herb species. (2) We identified richness as the number of species^34^. We preferred the number of total species recorded in this study area. If a study only reported the mean richness and standard error, we extracted the mean value. (3) If one publication reported data for more than one independent pair of secondary-primary, each pair was considered as an independent case study^35^. (4) When the same study was reported in different publications (different topics) by the same authors, we recorded the data only once. As a result, we extracted a total of 125 secondary-primary paired data. The locations of all studies included in this meta-analysis are shown in Fig. 1. The majority of locations were in the Changbai Mountains in Jilin Province, Qinling Mountains in Shaanxi Province, Ailao Mountains in Yunnan Province and Bawangling in Hainan Province. The sites were not spread evenly across China because there are few forests in the plains of east China and the Qinghai-Tibet Plateau of west China, even though these account for a large part of China’s land area.

### Statistical analyses

The studies varied substantially in the geographic regions and logging histories of data reported. We therefore sorted data by region and logging history^36^ (Fig. 2). The unit of analysis was a pair of secondary forests plant richness (*SR*) and primary forests plant richness (*PR*). The recovery (*R*) was the ratio calculated by equation (1):

1 $$R=\frac{SR}{PR}$$

For each pair, the natural log (ln*R*) was used to calculate the effect size. Because the type of the data was non-comparative binary odds, the variance (${v}_{{ln}R}$) and standard error (*SE*) of ln*R* ^37–39^ was estimated as follows:

2 $${v}_{lnR}=\frac{1}{SR}+\frac{1}{PR}$$

3 $$SE=\sqrt{{v}_{lnR}}$$

The analysis used a random-effect model since we aimed to estimate an overall effect and allowed variation between study effects^31, 40^. We used the random-effect model to calculate: the weights of corresponding comparisons, the effect size (recovery), 95% confidence interval (CI) and *p*-value for the null hypothesis that *SR*/*PR* = 1. We presented the recovery ratio as a percentage *R(%)* = 100% × *R*.

For heterogeneity analysis, Q-*df*, *p(Q-df)*, *I* ^2^ and *Tau* ^2^ were calculated^31^. Q-*df* presents the excess variation, which means that the part that will be attributed to differences in the true effects from study to study, while a significant *p(Q-df)* value provides evidence that the true effects vary. *I* ^2^ is the ratio of true heterogeneity to total variance across the observed effect estimates, and *Tau* ^2^ is the estimation for the variance of true effects.

Stata 14 (StataCorp LLC, Texas, USA) was applied in this analysis, with “metan” package^41^ for running the random-effect meta-analysis. To assess the publication bias and sensitivity of this analysis, we conducted additional analyses (Supplementary Information 1). The “metafunnel” package^42^ was used for funnel plots, “metabias” package^43^ for testing funnel-plot asymmetry and small study effects, as well as “metatrim” package^44^ for sensitivity analyses via “trim and fill” method.

## Electronic supplementary material

Supplementary Information

Supplementary Dataset
