# Supplementary material for: Recovery of woody plant species richness in secondary forests in China: a meta-analysis
Source: Sci Rep. 2017 Sep 6;7:10614. doi: 10.1038/s41598-017-10898-7 (PMC5587664; doi:10.1038/s41598-017-10898-7)
Supplement: Supplementary file 1 — Supplementary Information [file 41598_2017_10898_MOESM1_ESM.pdf]

Recovery of woody plant species richness in secondary forests in China: a meta-analysis

Xiaofei Liu[1], Xuehua Liu[1]*, Andrew Skidmore[2,3], Claude Garcia[4, 5]

1. State Key Joint Laboratory of Environmental Simulation and Pollution Control, and School of Environment, Tsinghua University, Beijing, 100084, China

2. ITC, University of Twente, 7500 AE Enschede, The Netherlands

3. Department of Environmental Science, Macquarie University, NSW 2109 Sydney, Australia

4. ForDev Group, Department of Environmental Systems Science, Swiss Federal Institute of Technology (ETH), CH-8092 Zurich, Switzerland

5. Research Unit Forests and Societies, Centre International de Recherche Agronomique pour le Développement (CIRAD), F-34392 Montpellier, France

*Corresponding author. Email: xuehua-hjx@mail.tsinghua.edu.cn.

Phone: +8601062794119. Fax: +860106279411

Supplementary Information 1

Publication bias identification and sensitivity analysis

A common concern with meta-analysis is testing for possible publication bias and to conduct sensitivity analysis to test the robustness of the results[1].

Firstly, we assessed our data graphically with the funnel plot portraying the effect size against standard error of each study (Supplementary Fig. S1). Funnel plots are

extensively used to examine publication bias and significant asymmetry around the mean effect size may indicate publication bias[2]. Visual assessment of our funnel plot did not suggest an obvious publication bias. Then we used Begg[3] and Egger[4] tests to quantify and test the funnel-plot asymmetry. The Begg test showed a non-significant bias (p=0.553) and the Egger regression test also showed a non-significant bias (p=0.796). Additionally the estimation of effect size was not biased by small sample sizes (p=0.796)[5]. Therefore the funnel plot, Begg and Egger tests all indicated the non-significant publication bias in our dataset.

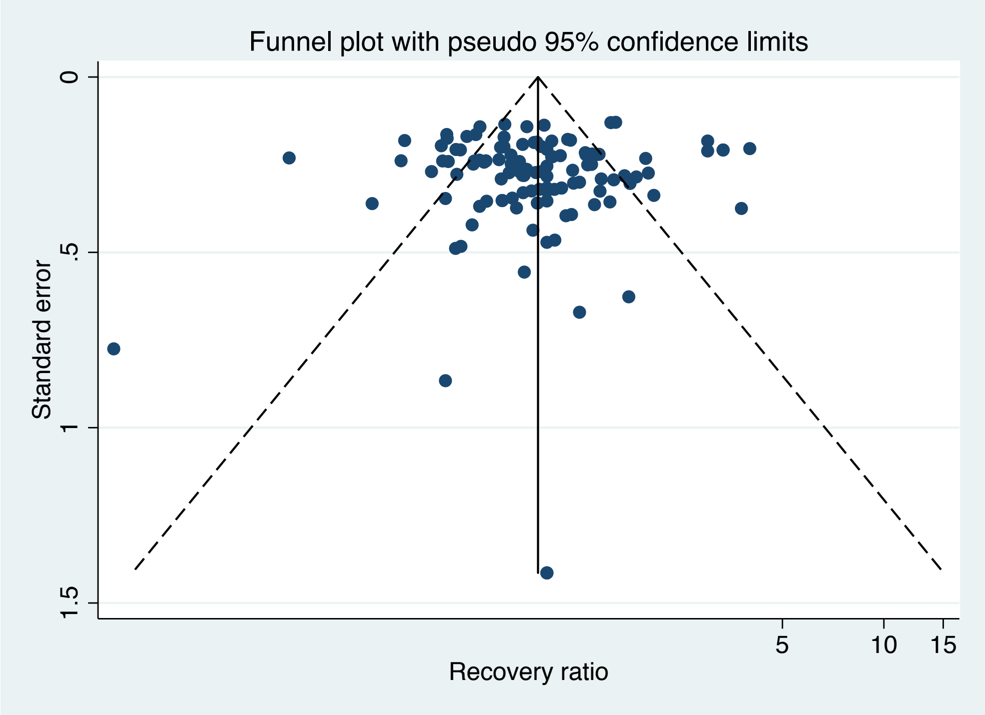

Supplementary Fig. S1 The funnel plot of effect size and standard error. The asymmetry around the mean effect size was not obvious (n=125).

Next, we conducted sensitivity analysis implementing "trim and fill" method. Trim and Fill uses an iterative procedure to remove the most extreme small study, re-compute the effect size and fill the estimated study at each iteration until the funnel plot is

symmetric about the new effect size[2]. However, in our dataset, trim and fill analysis did not identify any extreme small studies and did not remove or fill any studies (data unchanged). This indicated the robustness of our meta-analysis results.

References

1. Koricheva, J. & Gurevitch, J. Uses and misuses of meta-analysis in plant ecology. *J. Ecol.* **102**, 828-844 (2014).
2. Borenstein, M., Hedges, L. V., Higgins, J. P. T. & Rothstein, H. R. *Introduction to Meta-Analysis* (John Wiley & Sons, 2009).
3. Begg, C. B. & Mazumdar, M. Oprating characteritics of a bank correlation test for publication bias. *Biometrics* **50**, 1088-1101 (1994).
4. Egger, M., Smith, G. D., Schneider, M. & Minder, C. Bias in meta-analysis detected by a simple, graphical test. *BMJ* **315**, 629-634 (1997).
5. Harbord, R. M., Harris, R. J. & Sterne, J. A. C. Updated tests for small-study effects in meta-analyses. *Stata J.* **9**, 197-210 (2009).

Supplementary Information 2

The detailed information of the 55 publications extracted for this meta-analysis is in Supplementary Dataset.xls.

List of original 55 publications included in this meta-analysis

<u>Publications in English</u>

Bu, W., Zang, R., Ding, Y., 2014. Functional diversity increases with species diversity along successional gradient in a secondary tropical lowland rainforest. Tropical Ecology 55, 393-401.

Cai, H., Di, X., Chang, S.X., Jin, G., 2016. Stand density and species richness affect carbon storage and net primary productivity in early and late successional temperate forests differently. Ecological Research 31, 525-533.

Castroizaguirre, N., Chi, X., Baruffol, M., Tang, Z., Ma, K., Schmid, B., Niklaus, P.A., 2016. Tree diversity enhances stand carbon storage but not leaf area in a subtropical forest. Plos One 11, e0167771.

Chen, J., Rui, Y., Zhou, X., Ye, W., Liu, W., 2016. Determinants of the biodiversity patterns of ammonia-oxidizing archaea community in two contrasting forest stands. Journal of Soils and Sediments 16, 878-888.

Eichenberg, D., Trogisch, S., Huang, Y., He, S.J., Bruelheide, H., 2015. Shifts in

community leaf functional traits are related to litter decomposition along a secondary forest succession series in subtropical China. Journal of Plant Ecology 8(4), 401.

He, Z.B., Zhao, W.Z., Liu, H., Zhang, Z.H., 2010. Successional process of Picea crassifolia forest after logging disturbance in semiarid mountains: a case study in the Qilian Mountains, northwestern China. Forest Ecology & Management 260, 396-402.

Huang, Y., Ai, X., Yao, L., Zang, R., Ding, Y., Huang, J., Feng, G., Liu, J., 2015. Changes in the diversity of evergreen and deciduous species during natural recovery following clear-cutting in a subtropical evergreen-deciduous broadleaved mixed forest of central China. Tropical Conservation Science 8, 1033-1052.

Li, X.S., Liu, W.Y., Tang, C.Q., 2010. The role of the soil seed and seedling bank in the regeneration of diverse plant communities in the subtropical Ailao Mountains, Southwest China. Ecological Research 25, 1171-1182.

Liang, Y., He, X., Liang, S., Zhang, W., Chen, X., Feng, S., Su, Y., 2014. Community structure analysis of soil ammonia oxidizers during vegetation restoration in southwest China. Journal of Basic Microbiology 54, 180.

Lin, D., Jiang, Y., Zang, R., Wang, X., Long, W., Huang, J., Kang, Y., Xie, Z., 2016. Patterns of species diversity are not consistent between shifting cultivation in Bawangling Nature Reserves and selective logging in Diaoluoshan Nature Reserves, Hainan Island, China. Tropical Conservation Science 9, 584-606.

Long, W., Yang, X., Li, D., 2012. Patterns of species diversity and soil nutrients along a chronosequence of vegetation recovery in Hainan Island, South China. Ecological Research 27, 561-568.

Meng, J., Lu, Y., Lei, X., Liu, G., 2011. Structure and floristics of tropical forests and their implications for restoration of degraded forests of China's Hainan Island. Tropical Ecology 52, 177-191.

Mi, X., Swenson, N.G., Jia, Q., Rao, M., Feng, G., Ren, H., Bebber, D.P., Ma, K., 2016. Stochastic assembly in a subtropical forest chronosequence: evidence from contrasting changes of species, phylogenetic and functional dissimilarity over succession. Scientific Reports 6, 32596.

Mo, X.X., Shi, L.L., Zhang, Y.J., Zhu, H., Slik, J.W., 2013. Change in phylogenetic community structure during succession of traditionally managed tropical rainforest in southwest China. Plos One 8, e71464.

Song, P., Ren, H., Jia, Q., Guo, J., Zhang, N., Ma, K., 2015. Effects of historical logging on soil microbial communities in a subtropical forest in southern China. Plant and Soil 105, 1-12.

Tang, C.Q., Chiou, C.R., Lin, C.T., Lin, J.R., Hsieh, C.F., Tang, J.W., Su, W.H., Hou, X., 2013. Plant diversity patterns in subtropical evergreen broad-leaved forests of Yunnan and Taiwan. Ecological Research 28, 81-92.

Tang, C.Q., Hou, X., Gao, K., Xia, T., Duan, C., Fu, D., 2007. Man-made versus natural forests in Mid-Yunnan, Southwestern China: plant diversity and initial data on water and soil conservation. Mountain Research & Development 34, 242-249.

Tang, C.Q., Li, Y.H., Zhang, Z.Y., Hou, X., Hara, K., Tomita, M., He, L.Y., Li, X.S., 2015. Effects of management on vegetation dynamics and associated nutrient cycling in a karst area, Yunnan, SW China. Landscape and Ecological Engineering

11, 177-188.
Tang, C.Q., Li, Y.H., Zhang, Z.Y., Hurni, H., Dach, S.W.V., Zimmermann, A., 2016. Species diversity patterns in natural secondary plant communities and man-made forests in a subtropical mountainous karst area, Yunnan, SW China. Mountain Research & Development 30, 244-251.
Wang, K.B., Shao, R.X., Shangguan, Z.P., 2010. Changes in species richness and community productivity during succession on the Loess Plateau (China). Polish Journal of Ecology 58, 501-510.
Yuan, Z., Wang, S., Gazol, A., Mellard, J., Lin, F., Ye, J., Hao, Z., Wang, X., Loreau, M., 2016. Multiple metrics of diversity have different effects on temperate forest functioning over succession. Oecologia 182, 1-11.
Zhang, C., Jin, W., Gao, L., Zhao, X., 2014. Scale dependent structuring of spatial diversity in two temperate forest communities. Forest Ecology & Management 316, 110-116.
Zhu, W.Z., Song, C., Cai, X.H., Fei, H., Wang, J.X., 2009. Changes in plant species diversity along a chronosequence of vegetation restoration in the humid evergreen broad-leaved forest in the Rainy Zone of West China. Ecological Research 24, 315-325.

Publications in Chinese

Bao, W.K., Liu, Z.G., 2002. Community features of the primary and naturally secondary evergreen broad-leaved forests in MT. Wawu in Sichuan. China. Chinese Journal of Applied and Environmental Biology 8(2), 120-126.
Bu, W.S., 2013. The relationships between biodiversity and ecosystem functioning in natural tropical forests of Hainan Island, China. Dissertation, Chinese Academy of Forestry.
Chang, X.H., 2009. Study on ecosystem management of broad-leaved korean pine forest in Changbai Mountain. Dissertation, Beijing Forestry University.
Cui, J.W., 2006. Effects of anthropogenic disturbance on plant diversity and community structure of karst vegetation in stone-forest limestone region, Yunnan province. Dissertation, Chinese Academy of Sciences (Xishuangbanna Tropical Botanical Garden).
Du, Z., 2013. Study on stand structure during different succession stages in degraded spruce-fir forests of Changbai Mountains. Dissertation, Beijing Forestry University.
Feng, G., Ai, X.R., Yao, Liu, J., Huang, Y., Lin, Y., 2016. Dynamics of natural restoration of subtropical evengreen-deciduous broadleaved mixed forests in Southwest Hubei Province and influencing factors. Scientia Silvae Sinicae 52, 1-9.
Guo, Y.X., 2013. Characteristics and stability of betula albosinensis community in Qinling Mountains. Dissertation, Northwest Agriculture and Forestry University.
He, J.L., Cao, H.L., Zhang, Y.L., Ye, W.H., Li, W.J., Wu, L.F., 2009. Relationship between vegetation, landform and soil nutrients in karst forest of Mulun, Guangxi, China. Journal of Tropical and Subtropical Botany 17, 502-509.
Hu, J.Y., 2009. Study on the ecological characteristics of natural and secondary forest of fagus pashanica. Dissertation, Sichuan Agricultural University.

Hu, N., 2008. A study on plant functional groups and their dynamics in forest ecosystem on Funiu Mountain Nature Reserve. Dissertation, Henan University.

Huang, R., Wang, C., Yang, Z.J., Chen, S.G., 2011. Allocation of carbon storage in the arbor layer of young and old-growth evergreen broad-leaved forests in Wanmulin. Journal of Resources and Environment 06, 29-35.

Jiang, L.C., 2007. A study on the characteristics of degraded communities under different disturbances in Tiantong, Zhejiang Province, China. Dissertation, East China Normal University.

Jin, Y.H., Li, D.Q., Jiang, H.X., Liu, J., Li, G.H., 2006. Changes in species diversity of natural secondary forests after selective cutting disturbance in Changbai Mountain. Journal of Jilin Agricultural University 28, 35-39.

Li, S.F., 2011. Restoration ecology of monsoonal broad-leaved evergreen forest in Pu'er City, Southwest China. Dissertation, Chinese Academy of Forestry.

Li, X.M., 2014. Study on the tree diversity and stand stability of the broad-leaved Korean pine forest on the northern slope of Changbai mountain. Dissertation, Northeast Forestry University.

Liu, X.Z., Lu, Y.C., Zhou, Y.H., 2010. Dynamic changes of plant community structure and population niche in the recovery process of degenerated secondary forests. Chinese Journal of Ecology 29, 22-28.

Meng, J.H., 2009. Close-to-nature concept for restoration and management of tropical secondary forest on Hainan Island. Dissertation, Chinese Academy of Forestry.

Shi, J.N., Li, W.J., Meng, J.H., 2016. A comparison between tropical primary and secondary forests on Hainan Island. Chinese Journal of Applied and Environmental Biology 2, 271-276.

Sun, H.L., 2010a. China ecosystem location observation and research data set, forest ecosystem volume, Tibet Linzhi station 2001-2007. China Agriculture Press, Beijing.

Sun, H.L., 2010b. China ecosystem location observation and research data set, forest ecosystem volume, Yunnan Xishuangbanna station 1998-2006. China Agriculture Press, Beijing.

Sun, H.L., Han, S.J., 2012. China ecosystem location observation and research data set, forest ecosystem volume, Jilin Changbai Mountain station 2001-2008. China Agriculture Press, Beijing.

Sun, H.L., Zhang, M.Q., 2011. China ecosystem location observation and research data set, forest ecosystem volume, Guangdong Dinghu mountain station 1998-2008. China Agriculture Press, Beijing.

Sun, H.L., Zhang, Q.L., Gao, H.R., Ma, X.Z., 2011. China ecosystem location observation and research data set, forest ecosystem volume, Inner Mongolia Da Hinggan Mountains station 2006-2008. China Agriculture Press, Beijing.

Tang, J.M., 2008. Studies on restoration of montane mixed evergreen and deciduous broadleaved forests in Southwest Hubei Province. Dissertation, Beijing Forestry University.

Xia, F.C., 2007. Study on the plant biodiversity and spatial patterns of broad-leaved korean pine forest in Changbai Mountain. Dissertation, Beijing Forestry University.

Yang, J., 2007. The species diversity and stability of sub-alpine plantation communication in the upper reach of Minjiang River. Dissertation, Sichuan

Agricultural University.
Yang, W.Y., 2010. Community structure and natural regeneration of natural yunnan pine forest in Middle Yunnan, China. Dissertation, Chinese Academy of Forestry.
Yuan, S.Y., 2010. Evaluation of current forest management models in Xiaolongshan Gansu Province. Dissertation, Chinese Academy of Forestry.
Zeng, Z.X., Wang, K.L., Liu, X.L., Zeng, F.P., 2016. Stoichiometric characteristics of live fresh leaves and leaf litter from typical plant communities in a karst region of northwestern Guangxi, China. Acta Ecologica Sinica 36, 1907-1914.
Zhang, C.H., Zhu, J.J., Yan, Q.L., Yu, L.Z., 2009. Comparison of plant species diversity and factors influencing species diversity of primeval and secondary forests on the northern slope of Changbai Mountains, China. Journal of Northeast Forestry University 37, 52-55.
Zhao, Z.H., 2009. Evaluating forest naturalness based on stand state characteristics. Dissertation, Chinese Academy of Forestry.
Zhao, Z.H., Hui, G.Y., Yuan, S.Y., Liu, W.Z., Wang, R.X., 2009. Spatial structure characteristic of quercus aliena var. acuteserrata natural forest in Xiaolongshan. Scientia Silvae Sinicae 45, 1-6.
